# Glenohumeral Hydrodistension for Postoperative Stiffness After Arthroscopic Primary Rotator Cuff Repair

**DOI:** 10.1177/23259671221104505

**Published:** 2022-06-14

**Authors:** Ryan H. Barnes, Anthony V. Paterno, Feng-Chang Lin, Jingru Zhang, David Berkoff, R. Alexander Creighton

**Affiliations:** † Department of Orthopaedics, University of North Carolina Hospitals, Chapel Hill, North Carolina, USA.; ‡ Department of Biostatistics, University of North Carolina, Chapel Hill, North Carolina, USA.; *Investigation performed at the University of North Carolina Hospitals, Chapel Hill, North Carolina, USA*

**Keywords:** rotator cuff repair, hydrodistension, glenohumeral hydrodistension, postoperative stiffness, stiffness

## Abstract

**Background:**

Postoperative stiffness is a known complication after rotator cuff repair (RCR). Glenohumeral hydrodistension (GH) has been a treatment modality for shoulder pathology but has not been used to treat postoperative stiffness after RCR.

**Purpose/Hypothesis:**

The purpose of this study was to identify the risk factors for postoperative stiffness after RCR and review outcomes after treatment with GH. Our hypotheses were that stiffness would be associated with diabetes and hyperlipidemia and correlated with the tendons involved and that patients with stiffness who underwent GH would have significant improvement in range of motion (ROM).

**Study Design:**

Case series; Level of evidence, 4.

**Methods:**

Included were 388 shoulders of patients who underwent primary RCR by a single surgeon between 2015 and 2019. Shoulders with revision RCRs were excluded. Patient characteristics, medical comorbidities, and perioperative details were collected. A total of 40 shoulders with postoperative stiffness (10.3%) received GH injectate of a 21-mL mixture (15 mL of sterile water, 5 mL of 0.5% ropivacaine, and 1 mL of triamcinolone [10 mg/mL]). The primary outcome measure was ROM in forward flexion, internal rotation, external rotation, and abduction. Statistical tests were performed using analysis of variance.

**Results:**

Patients with diabetes had significantly decreased internal rotation at final follow-up after RCR as compared with patients without diabetes. GH to treat stiffness was performed most commonly between 1 and 4 months after RCR (60%), and patients who received GH saw statistically significant improvements in forward flexion, external rotation, and abduction after the procedure. Patients with hyperlipidemia had the most benefit after GH. Among those undergoing concomitant procedures, significantly more patients who had open subpectoral biceps tenodesis underwent GH. Patients who underwent subscapularis repair or concomitant subacromial decompression had significant improvement in ROM after GH. Only 1 patient who received GH underwent secondary surgery for resistant postoperative stiffness.

**Conclusion:**

Patients with diabetes had increased stiffness. Patients with a history of hyperlipidemia or concomitant open subpectoral biceps tenodesis were more likely to undergo GH for postoperative stiffness. Patients who underwent subscapularis repair demonstrated the most improvement in ROM after GH. After primary RCR, GH can increase ROM and is a useful adjunct for patients with stiffness to limit secondary surgery.

Postoperative shoulder stiffness is a known complication after rotator cuff repair (RCR), with a reported incidence of 4.9%.^8,14^ The condition has been attributed to intra-articular contractures and to adhesion of the tendons.^1^ Risk factors for postoperative stiffness after RCR include decreased preoperative range of motion (ROM), workers’ compensation cases, and diabetes mellitus.^1^ Preoperative stiffness can have a significant effect on the early postoperative recovery period, outcomes, and pain scores.^3^ Treatment of postoperative stiffness typically consists of nonoperative management with oral nonsteroidal anti-inflammatory drugs (NSAIDs) and physical therapy.^10^ However, arthroscopic capsular release is performed in an estimated 3.3% of cases of postoperative stiffness.^4^ Millican et al^12^ found postoperative stiffness to be beneficial, as a way to protect the repair, and to ultimately resolve within 5 years.

There are limited data on alternative nonsurgical treatment modalities for postoperative stiffness, such as intra-articular steroid injections and glenohumeral hydrodistension (GH), 2 treatments that have been shown to be effective in the treatment of adhesive capsulitis. Studies have demonstrated that GH used as treatment for adhesive capsulitis allowed for earlier pain relief, significant improvements in shoulder scores, and greater improvement in ROM when compared with intra-articular steroid injections.^6,7,11,13,15–18,21^ Yet, other studies have shown that although GH can be effective, it has no clinical superiority as compared with intra-articular steroid injections.^9,19,22^ When used in combination to treat adhesive capsulitis, GH and intra-articular steroid injections have been shown to expedite pain control and ROM improvement.^2^ GH can be used for multiple techniques and approaches to the shoulder.^5^ Watson et al^20^ demonstrated improved outcomes and pain thresholds when using GH to address rotator cuff pathology. However, it has not been shown as a treatment modality for postoperative stiffness.

The purpose of this study was to determine the risk factors for postoperative stiffness after RCR and the outcomes of patients who underwent GH. Our hypotheses were that (1) stiffness would be associated with diabetes and hyperlipidemia and correlated with the tendons involved and (2) patients with stiffness who underwent GH would have significant improvement in ROM.

## Methods

This study was determined to be exempt from institutional review board approval. We retrospectively reviewed patients who underwent arthroscopic RCR (identified via Current Procedural Terminology code 29827) performed by the senior author (R.A.C.) at our high-volume academic medical center between 2015 and 2019. Excluded were patients who had prior ipsilateral shoulder surgery, whether open shoulder surgery, arthroscopic debridement, labral repair, or prior RCR.

**Figure 1.**
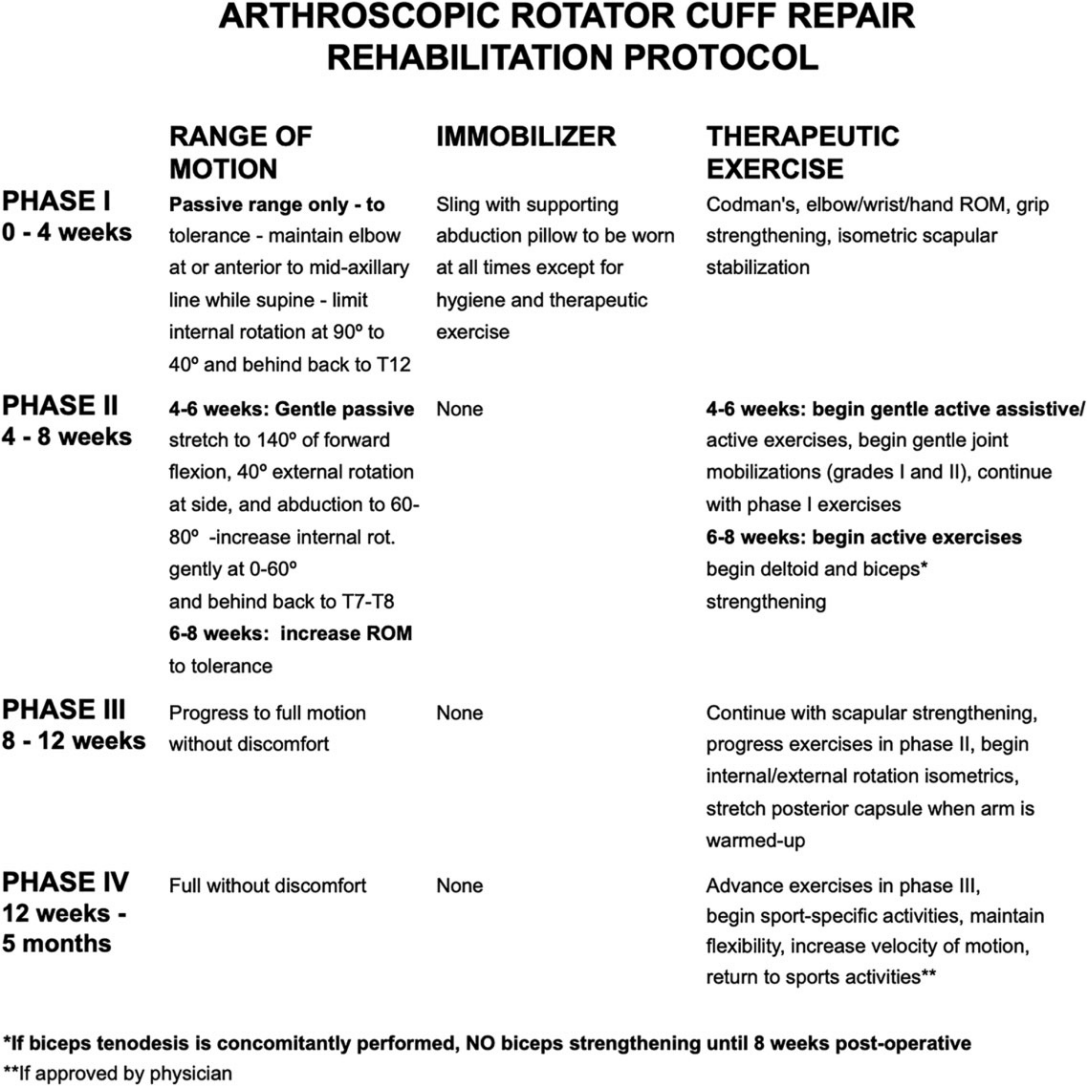
Standardized rehabilitation protocol for rotator cuff repair until 5 months postoperatively. ROM, range of motion; rot, rotation.

All patients underwent double-row RCR. Concomitant procedures included subacromial decompression with acromioplasty, distal clavicle excision, and biceps tenodesis. Indications to perform acromioplasty were based on intraoperative findings consistent with impingement signs, such as fraying of the coracoacromial ligament. Distal clavicle excision was based primarily on preoperative symptoms and physical examination findings, including pain with cross-body shoulder adduction and symptomatic acromioclavicular osteoarthritis. Biceps tenodesis was performed per a combination of physical examination findings, such as tenderness to palpation in the bicipital groove and positive O’Brien test result, as well as intraoperative findings of unstable superior labrum with tenosynovitis in the groove. Results were stored within a secure database. All patients underwent a standardized postoperative rehabilitation protocol (Figure 1).

### Glenohumeral Hydrodistension

Shared decision making between the patient and the senior surgeon (R.A.C.) was made before performing GH. The decision to proceed with GH was based on patient-reported symptoms and a lack of progress or plateauing with physical therapy and was determined on a case-by-case basis by the senior surgeon. GH was performed in a standardized fashion for each patient in the office setting without anesthesia using a standard linear musculoskeletal ultrasound probe (5-15 MHz). Using the probe, the surgeon identified the posterior humerus, glenoid, and labrum. The injectate is a 21-mL mixture, per the provider’s protocol, that consists of 15 mL of sterile water, 5 mL of 0.5% ropivacaine, and 1 mL of triamcinolone (10 mg/mL). The area was prepared, and the path to the posterior joint was anesthetized using 2 mL of 1% lidocaine. Using ultrasound throughout the procedure to ensure proper needle placement, the surgeon injected the glenohumeral joint via a 6.35-cm 22-gauge needle, visualizing the capsular distension until the entire volume was injected into the glenohumeral joint capsule.

### Study Variables

Charts of all patients were reviewed via the electronic medical record, and data were collected for basic patient demographic information (age at the time of surgery [grouped by 5-year increments], sex, body mass index [BMI]) as well as medical comorbidities at the time of surgery and hand dominance. Also recorded were tendon involvement (based on preoperative magnetic resonance imaging scans), surgical findings and surgical procedures (tendons repaired, number of anchors used, and concomitant procedures), and details of GH treatment (whether and when it was performed and ROM changes after the procedure).

ROM was measured by the senior surgeon pre- and postoperatively and after GH based on a clinical examination that was visually recorded. ROM was measured via active motion. External rotation was measured at the patient’s side. Internal rotation was measured via spinal level using a scale with the following increments and numeric scores: sacrum (1), L5 (2), L1-L4 (3), T7-T12 (4), T1-T6 (5), and C1-C7 (6). ROM was obtained on a patient-by-patient basis as determined fit by the senior surgeon; thus, not every patient received ROM assessments for all movements.

### Statistical Analysis

Summary statistics were used to describe the demographic and clinical features of participating patients (mean ± SD, median and interquartile range, and frequencies and proportions). The primary outcome was ROM in forward flexion, abduction, internal rotation, and external rotation, which was expressed as mean and standard deviation. We compared the primary outcome between patients with and without comorbidities and according to the tendons repaired and concomitant procedures. We also compared ROM between patients who received GH treatment and those who did not. Comparisons across treatment groups were based on chi-square test of homogeneity for categorical variables and analysis of variance or Kruskal-Wallis test (for medians) for continuous variables. *P* < .05 was considered the threshold for statistically significant differences. Statistical analyses were performed using SAS Version 9.4 (SAS Institute).

## Results

**Figure 2.**
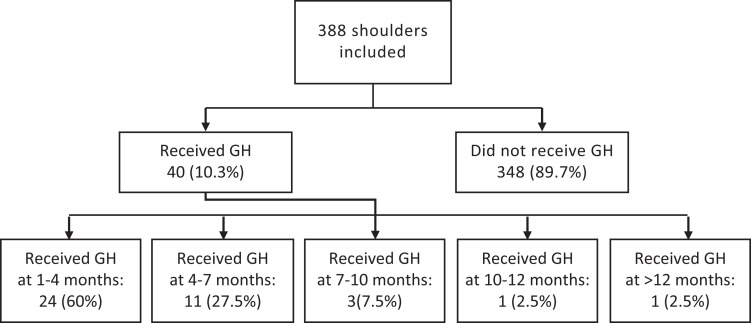
CONSORT (Consolidated Standards of Reporting Trials) diagram of the study enrollment process and the number of months after surgery when the glenohumeral hydrodistension (GH) was provided.

Of 423 shoulders eligible for the study, 35 were excluded: 25 with revision RCRs and 10 that underwent superior capsular reconstruction. Thus, 388 shoulders met the inclusion criteria (Figure 2). Of those, 53% were male (Table 1). The mean BMI at the time of surgery was 29.8 ± 6.6. Surgery was most commonly performed in patients aged ≥65 years (32.1%), followed by those 55 to 59 years old (19.4%). Overall, 62% of RCRs were performed on right shoulders, with the majority of patients being right-hand dominant (64%). The mean postoperative follow-up period was 158 days.

**Table 1:** Patient Characteristics Overall and According to Hydrodistension Treatment Group^*a*^

		Underwent Hydrodistension		
	Total (N = 388)	No (n = 348)	Yes (n = 40)	*P* Value
Sex				.29
Female	183 (47)	161 (46)	22 (55)	
Male	205 (53)	187 (54)	18 (45)	
Body mass index				.36
Mean ± SD	29.8 ± 6.6	30.0 ± 6.7	28.7 ± 6.3	
Median (IQR)	28.9 (25.1-33.9)	29.0 (25.4-33.9)	28.3 (24.1-32.9)	
Side of rotator cuff repair				.48
Right	242 (62)	215 (62)	27 (68)	
Left	146 (37)	133 (38)	13 (33)	
Arm dominance				.25
Right	109 (92)	95 (91)	14 (100)	
Left	9 (8)	9 (9)		
Dominant arm involved				.86
Yes	76 (64)	68 (64)	8 (67)	
No	42 (36)	38 (36)	4 (33)	

*^a^* Values are expressed as No. (%) of shoulders unless otherwise indicated. IQR, interquartile range.

Of those shoulders that developed postoperative stiffness, 40 (10.3%) underwent GH. The GH procedure was most commonly performed from 1 to 4 months postoperatively (60%) (Figure 2). Of the 40 patients who underwent GH, 55% were female, 68% had right-side RCR, and the average BMI was 28.7 ± 6.3. None of these patient factors was significantly associated with the decision to perform GH (Table 1).

Overall, the most prevalent medical comorbidities were hypertension (38% of patients), hyperlipidemia (22%), and diabetes (15%) (Table 2). Hemoglobin A1c was typically well controlled (43.6%) (<6.5). However, of the patients with diabetes, 19% had poor control (>8.5). The only medical comorbidity that reached statistical significance in relation to undergoing GH was hyperlipidemia (*P* = .03). The only concomitant surgical procedure that reached statistical significance in relation to undergoing GH was open subpectoral biceps tenodesis, performed via a 2- to 3-cm incision (*P* = .046) (Table 3).

**Table 2:** Medical Comorbidities According to Hydrodistension Group^*a*^

		Underwent Hydrodistension		
	Total	No	Yes	*P* Value
Diabetes mellitus				.65
Yes	58 (15)	53 (15)	5 (13)	
No	330 (85)	295 (85)	35 (88)	
Hypertension				.72
Yes	146 (38)	132 (38)	14 (35)	
No	242 (62)	216 (62)	26 (65)	
Hyperlipidemia				**.03**
Yes	85 (22)	71 (20)	14 (35)	
No	303 (78)	277 (80)	26 (65)	

*^a^* Values are expressed as No. (%) of shoulders. Bold *P* value indicates statistically significant difference between groups (*P* < .05).

**Table 3:** Rotator Cuff Repair Characteristics and Concomitant Procedures According to Hydrodistension Group*^a^*

		Underwent Hydrodistension		
	Total	No	Yes	*P* Value
No. of anchors used				
Mean ± SD	3.1 ± 1.5	3.0 ± 1.5	3.3 ± 1.7	.39
Median (IQR)	3.0 (2.0-4.0)	3.0 (2.0-4.0)	3.0 (2.0-5.0)	
No. of tendons involved^*b*^				
Mean ± SD	1.7 ± 0.7	1.7 ± 0.7	1.6 ± 0.8	.84
Median (IQR)	2.0 (1.0-2.0)	2.0 (1.0-2.0)	1.0 (1.0-2.0)	
Supraspinatus involved				.19
Yes	320 (82)	284 (82)	36 (90)	
No	68 (18)	64 (18)	4 (10)	
Infraspinatus involved				.76
Yes	166 (43)	148 (43)	18 (45)	
No	222 (57)	200 (57)	22 (55)	
Subscapularis involved				.79
Yes	74 (19)	67 (19)	7 (18)	
No	314 (81)	281 (81)	33 (83)	
Teres minor involved				.40
Yes	6 (2)	6 (2)		
No	382 (98)	342 (98)	40 (100)	
Concomitant subacromial decompression				.13
Yes	199 (51)	174 (50)	25 (63)	
No	189 (49)	174 (50)	15 (38)	
Concomitant distal clavicle excision				.14
Yes	60 (15)	57 (16)	3 (8)	
No	328 (85)	291 (84)	37 (93)	
Concomitant open biceps tenodesis				**.046**
Yes	214 (55)	186 (53)	28 (70)	
No	174 (45)	162 (47)	12 (30)	

*^a^* Values are expressed as No. (%) of shoulders unless otherwise indicated. Bold *P* value indicates statistically significant difference between groups (*P* < .05). IQR, interquartile range.

*^b^* According to preoperative magnetic resonance imaging scan.

In patients who received GH, there was a statistically significant improvement in ROM for forward flexion (*P* < .001), external rotation (*P* < .001), and abduction (*P* = .01) as compared with pretreatment (Table 4).

**Table 4:** Difference in Range of Motion From Before to After Glenohumeral Hydrodistension Treatment (n = 40)*^a^*

	Range of Motion, deg		
	Pretreatment*^b^*	Posttreatment	*P* Value
Forward flexion	116.2 ± 31.2	154.2 ± 20.5	**<.001**
External rotation	31.3 ± 18.7	49.5 ± 13.9	**<.001**
Internal rotation	3.0 ± 1.2	3.3 ± 0.9	.2963
Abduction	97.7 ± 31.2	140.0 ± 41.9	**.01**

*^a^* Bold *P* values indicate statistically significant difference between groups (*P* < .05).

*^b^* Last recorded maximal motion in each plane before receiving glenohumeral hydrodistension.

Regarding the effect of medical comorbidities on ROM, patients with diabetes demonstrated a statistically significant decrease in final postoperative internal rotation compared with patients without diabetes (*P* = .02) (Appendix Table A1). There was no difference in ROM postoperatively between patients with and without hyperlipidemia who did not undergo GH. However, when compared with patients without hyperlipidemia who underwent GH for postoperative stiffness, there was a statistically significant decrease in ROM for patients with hyperlipidemia in forward flexion, external rotation, and abduction but not internal rotation (Appendix Table A2).

In terms of the effect of involved tendons in the RCR, for patients with subscapularis repairs who subsequently underwent GH, there was a statistically significant increase in ROM in forward flexion, external rotation, and internal rotation (Appendix Table A2). For the effect of concomitant surgical procedures, patients who underwent GH had significantly reduced external rotation if they had undergone a subacromial decompression with RCR as compared with if they had not undergone a subacromial decompression (Appendix Table A3).

Only 1 of the 40 patients who received GH subsequently underwent an additional procedure for stiffness.

## Discussion

Our study demonstrates the efficacy of GH for postoperative stiffness after RCR as reflected by increased ROM. We believe that GH is an effective adjuvant nonoperative treatment modality for postoperative stiffness and that 10.3% of all shoulders with RCRs undergoing GH is a clinically compelling number. The incidence of postoperative stiffness was higher than that seen in previous studies (4.9%).^8,14^ We attribute this difference to having a lower threshold of defining stiffness.

Understanding risk factors and outcomes around RCR is critical for identifying and treating patients with postoperative stiffness. We noted the association between subscapularis repair and statistically significant improvement in ROM after GH; however, subscapularis repair was not associated with undergoing GH. This suggests that although subscapularis repair did not predict the need for GH in our study, patients who had subscapularis repair and received GH had better ROM than did the rest of the GH cohort. We attribute this finding to potential disruptions in biomechanics and increased pain secondary to small suboptimal tendon disruption during RCR, which was addressed in only the subscapularis repair cohort. Additional studies are required to investigate this observation further.

With regard to patient selection for receiving GH, the senior surgeon discussed risks and benefits with each patient before making a recommendation. This included patients with diabetes and the known risk factors associated with receiving a corticosteroid injection. Generally, there was no reluctance to perform GH unless the patient had issues with prior corticosteroid injection.

We believe that recognizing open subpectoral biceps tendon tenodesis as a risk factor for undergoing GH is clinically meaningful given that such a high proportion of all RCRs have concomitant open biceps tenodesis. We surmise that even though an open subpectoral biceps tenodesis is extra-articular, it can still affect postoperative pain, ROM, and stiffness. Completion of the tenodesis requires a 2- to 3-cm incision and can lead to scar tissue along the pectoralis tendon and increased postoperative pain. Thus, an intra-articular procedure such as GH can provide a therapeutic benefit. We speculate that this association may be due to the increased potential adhesions secondary to an additional procedure outside the glenohumeral joint; however, further studies are required to be confident about the cause. We attribute the benefit seen in patients to a combination of the capsular distension and the corticosteroid. It is also important to discuss with patients the possible risks of this procedure: inhibition of tendon healing from the steroid, retear from distension, and complications related to receiving a corticosteroid injection.

Our study contributes to the literature by confirming results of postoperative stiffness after RCR. Huberty et al^8^ described the rate of postoperative stiffness and related patient factors; however, our study builds on this by describing the rate of undergoing GH as well as the various patient and surgical risk factors that could contribute. After GH, only 1 patient underwent a secondary procedure for lysis of adhesions (0.25%), which is a lower rate than that in previous literature.^4^ [Bibr bibr4-23259671221104505]

### Limitations

There are several limitations to this study. First, all of the study patients underwent RCR from a single sports medicine orthopaedic surgeon. This allows for some bias toward operative tendencies and clinical decision making. There was not a strict definition of stiffness or decision of when to perform GH; rather, this was determined on a case-by-case basis with an in-depth discussion between the senior surgeon and patient. Also, the majority of GH procedures were performed within 1 to 4 months postoperatively, and it is difficult to determine whether these patients would have had improved postoperative ROM without GH with continued physical therapy alone. Patients did not receive postoperative imaging to determine if GH treatment led to more failed repairs. Future studies could include a case-control group of patients of the same sex and age and postoperative time to compare outcomes.

Without a control group, it is challenging to delineate whether hydrodistention with or without triamcinolone would provide different results in terms of ROM. Stiffness could also represent postoperative healing of the repair and be a natural part of the healing process. Although the data identified possible connections among ROM, medical comorbidities, and surgical findings, it is impossible to know whether they are truly clinically meaningful. Another limitation is that there was no breakdown of comorbidities by time after RCR for GH; neither was there any significance in terms of which comorbidities presented earlier versus later. Likewise, we did not perform multivariate and power analyses, which could lead to some findings becoming significant with a larger number of patients. In addition, 1 mL of triamcinolone was present within the injectate; thus, the improved ROM could be attributed to an intra-articular steroid injection.

Other limitations include lack of patient-reported outcome measures as well as no assessment of pain levels postprocedure. Finally, the mean follow-up time after RCR was 182days. Future studies could look at longer-term follow-up.

## Conclusion

We found the risk factors for stiffness after RCR to include diabetes mellitus and significantly decreased postoperative internal rotation, while hyperlipidemia and concomitant open subpectoral biceps tenodesis were statistically associated with receiving GH. Patients who received GH saw significant improvement in forward flexion, external rotation, and abduction ROM. Patients who underwent concomitant subacromial decompression had statistically significant improvement in ROM after GH. Findings indicated that GH is a useful adjunct for patients with stiffness after primary RCR to limit secondary surgery.

## Appendix

**Table A1:** Postoperative ROM (All Shoulders) vs Posttreatment ROM (GH Cases) According to Medical Comorbidities*^a^*

	Comorbidity		*P* Value
	Diabetes Mellitus (n = 58)	No Diabetes Mellitus (n = 330)	
Postoperative			
FF	140.9 ± 34.1	148.1 ± 28.8	.12
ER	48.9 ± 14.3	50.0 ± 16.2	.67
IR	3.0 ± 0.9	3.4 ± 0.9	**.020**
Ab	105.8 ± 36.6	117.1 ± 36.4	.30
Posttreatment^*b*^			
FF	146.3 ± 23.3	157.7 ± 18.0	.11
ER	50.0 ± 16.0	51.8 ± 12.6	.71
IR	2.7 ± 1.2	3.3 ± 0.8	.077
Ab	95.0 ± 7.1	142.0 ± 32.9	.081
	Hypertension (n = 146)	No Hypertension (n = 242)	
Postoperative			
FF	147.6 ± 29.1	146.6 ± 30.2	.77
ER	48.8 ± 15.6	50.6 ± 16.1	.34
IR	3.3 ± 1.0	3.3 ± 0.9	.96
Ab	117.8 ± 36.9	114.0 ± 36.4	.63
Posttreatment^*b*^			
FF	151.2 ± 20.1	159.5 ± 17.6	.087
ER	52.6 ± 12.9	51.0 ± 13.2	.64
IR	3.1 ± 0.9	3.4 ± 0.8	.20
Ab	115.0 ± 37.0	143.8 ± 32.0	.19
	Hyperlipidemia (n = 85)	No Hyperlipidemia (n = 303)	
Postoperative			
FF	148.8 ± 31.3	146.4 ± 29.2	.54
ER	49.9 ± 14.3	49.8 ± 16.5	.98
IR	3.3 ± 1.0	3.3 ± 0.9	.79
Ab	103.9 ± 34.9	119.5 ± 36.4	.077
Posttreatment^*b*^			
FF	148.3 ± 21.8	159.3 ± 16.9	**.035**
ER	45.0 ± 13.4	54.2 ± 12.0	**.010**
IR	3.1 ± 1.0	3.3 ± 0.8	.34
Ab	93.3 ± 5.8	147.8 ± 29.1	**.011**
	Hypothyroid (n = 23)	No Hypothyroid (n = 365)	
Postoperative			
FF	157.9 ± 23.5	146.3 ± 30.0	.099
ER	50.0 ± 14.9	49.8 ± 16.0	.96
IR	3.6 ± 0.7	3.3 ± 0.9	.26
Ab	142.1 ± 26.4	113.3 ± 36.4	**.043**
Posttreatment^*b*^			
FF	173.3 ± 5.8	155.4 ± 18.9	.11
ER	63.3 ± 5.8	51.0 ± 13.0	.11
IR	3.3 ± 0.6	3.2 ± 0.9	.86
Ab	170.0 ± 0.0	130.9 ± 34.8	.31

*^a^* Values are presented as mean ± SD. Bold *P* values indicate statistically significant difference between groups (*P* < .05). Ab, abduction; ER, external rotation; FF, forward flexion; GH, glenohumeral hydrodistension; IR, internal rotation; ROM, range of motion.

*^b^* For this comparison, n = 40 shoulders.

**Table A2:** Postoperative ROM (All Shoulders) vs Posttreatment ROM (GH Cases) According to Tendons Repaired*^a^*

	Tendon Repaired		*P* Value
	Supraspinatus Repair (n = 330)	No Supraspinatus Repair (n = 58)	
Postoperative			
FF	147.4 ± 29.9	142.6 ± 27.5	.46
ER	50.2 ± 16.0	45.7 ± 14.4	.19
IR	3.3 ± 0.9	3.1 ± 1.1	.41
Ab	115.7 ± 36.7	113.3 ± 36.7	.88
Posttreatment*^b^*			
FF	156.1 ± 19.4	158.0 ± 13.0	.83
ER	50.9 ± 12.9	60.0 ± 12.2	.13
IR	3.3 ± 0.8	2.8 ± 1.3	.23
Ab	134.2 ± 35.0	NA (n = 0)	NA
	Infraspinatus Repair (n = 171)	No Infraspinatus Repair (n = 217)	
Postoperative			
FF	146.2 ± 30.4	147.7 ± 29.2	.64
ER	49.4 ± 15.9	50.2 ± 16.0	.63
IR	3.2 ± 0.9	3.4 ± 0.9	.12
Ab	118.2 ± 38.3	113.1 ± 35.0	.51
Posttreatment*^b^*			
FF	153.2 ± 22.5	158.6 ± 15.5	.26
ER	50.4 ± 16.0	52.6 ± 10.1	.51
IR	3.3 ± 0.8	3.2 ± 0.9	.78
Ab	135.7 ± 34.1	132.0 ± 40.2	.87
	Subscapularis Repair (n = 82)	No Subscapularis Repair (n = 306)	
Postoperative			
FF	146.4 ± 31.6	147.2 ± 29.2	.84
ER	48.9 ± 15.0	50.1 ± 16.2	.56
IR	3.1 ± 0.9	3.3 ± 0.9	.13
Ab	114.2 ± 39.1	115.8 ± 36.0	.86
Posttreatment*^b^*			
FF	159.8 ± 16.1	140.8 ± 22.7	**.001**
ER	53.7 ± 10.9	41.8 ± 17.8	**.005**
IR	3.4 ± 0.8	2.4 ± 1.0	**.005**
Ab	138.2 ± 33.7	90.0 ± 0.0	.20
	Teres Minor Repair (n = 10)	No Teres Minor Repair (n = 378)	
Postoperative			
FF	138.3 ± 39.1	147.3 ± 29.5	.38
ER	56.7 ± 7.1	49.6 ± 16.1	.19
IR	3.0 ± 0.0	3.3 ± 0.9	.47
Ab	52.5 ± 10.6	116.9 ± 35.6	**.013**
Posttreatment*^b^*			
FF	NA	156.3 ± 18.9	NA
ER	NA	51.6 ± 13.0	NA
IR	NA	3.2 ± 0.9	NA
Ab	NA	134.2 ± 35.0	NA
	Single Tendon Involved (n = 158)	Multiple Tendons Involved (n = 190)	
Postoperative			
FF	148.4 ± 29.8	146.4 ± 29.9	.55
ER	50.4 ± 16.3	49.9 ± 15.7	.79
IR	3.4 ± 0.9	3.2 ± 0.9	.080
Ab	111.0 ± 35.9	119.9 ± 37.4	.26
Posttreatment*^b^*			
FF	160.6 ± 13.4	151.6 ± 22.8	.062
ER	53.2 ± 9.4	49.3 ± 16.0	.25
IR	3.4 ± 0.7	3.2 ± 0.9	.42
Ab	142.5 ± 37.7	130.0 ± 35.5	.58

*^a^* Values are presented as mean ± SD. Bold *P* values indicate statistically significant difference between groups (*P* < .05). Ab, abduction; ER, external rotation; FF, forward flexion; GH, glenohumeral hydrodistension; IR, internal rotation; NA, not applicable; ROM, range of motion.

*^b^* For this comparison, n = 40 shoulders.

**Table A3:** Postoperative ROM (All Shoulders) vs Posttreatment ROM (GH Cases) According to Concomitant Procedures*^a^*

	Concomitant Procedure		*P* Value
	Subacromial Decompression (n = 199)	No Subacromial Decompression (n = 189)	
Postoperative			
FF	146.9 ± 29.8	147.2 ± 29.8	.94
ER	49.7 ± 15.8	50.0 ± 16.2	.89
IR	3.3 ± 1.0	3.4 ± 0.8	.45
Ab	116.9 ± 37.2	112.9 ± 35.5	.63
Posttreatment*^b^*			
FF	152.4 ± 20.6	161.5 ± 15.1	.058
ER	48.1 ± 14.1	56.3 ± 9.7	**.011**
IR	3.1 ± 0.9	3.4 ± 0.7	.16
Ab	127.8 ± 38.7	153.3 ± 5.8	.29
	Distal Clavicle Excision (n = 60)	No Distal Clavicle Excision (n = 328)	
Postoperative			
FF	152.2 ± 24.7	146.0 ± 30.6	.18
ER	51.8 ± 11.6	49.4 ± 16.6	.34
IR	3.1 ± 1.1	3.3 ± 0.9	.20
Ab	121.3 ± 35.4	114.3 ± 36.8	.49
Posttreatment*^b^*			
FF	161.1 ± 17.6	155.5 ± 19.1	.41
ER	55.6 ± 16.7	50.9 ± 12.3	.33
IR	3.1 ± 1.1	3.3 ± 0.8	.74
Ab	116.7 ± 46.2	140.0 ± 31.6	.34
	Open Biceps Tenodesis (n = 214)	No Open Biceps Tenodesis (n = 174)	
Postoperative			
FF	149.1 ± 28.8	143.8 ± 31.0	.12
ER	50.9 ± 16.5	48.3 ± 14.9	.16
IR	3.3 ± 0.9	3.3 ± 0.9	.69
Ab	116.1 ± 34.3	114.6 ± 40.3	.85
Posttreatment*^b^*			
FF	155.7 ± 20.5	157.5 ± 15.2	.72
ER	49.8 ± 14.1	55.5 ± 9.4	.10
IR	3.2 ± 0.9	3.3 ± 0.9	.71
Ab	132.2 ± 36.7	140.0 ± 36.1	.76

*^a^* Values are presented as mean ± SD. Bold *P* value indicates statistically significant difference between groups (*P* < .05). Ab, abduction; ER, external rotation; FF, forward flexion; GH, glenohumeral hydrodistension; IR, internal rotation; ROM, range of motion.

*^b^* For this comparison, n = 40 shoulders.
